# Novelty or Surprise?

**DOI:** 10.3389/fpsyg.2013.00907

**Published:** 2013-12-11

**Authors:** Andrew Barto, Marco Mirolli, Gianluca Baldassarre

**Affiliations:** ^1^School of Computer Science, University of Massachusetts AmherstAmherst, MA, USA; ^2^Istituto di Scienze e Tecnologie della Cognizione, Consiglio Nazionale delle RicercheRome, Italy

**Keywords:** novelty, surprise, intrinsic motivation, novelty detection, expectation

## Abstract

Novelty and surprise play significant roles in animal behavior and in attempts to understand the neural mechanisms underlying it. They also play important roles in technology, where detecting observations that are novel or surprising is central to many applications, such as medical diagnosis, text processing, surveillance, and security. Theories of motivation, particularly of intrinsic motivation, place novelty and surprise among the primary factors that arouse interest, motivate exploratory or avoidance behavior, and drive learning. In many of these studies, novelty and surprise are not distinguished from one another: the words are used more-or-less interchangeably. However, while undeniably closely related, novelty and surprise are very different. The purpose of this article is first to highlight the differences between novelty and surprise and to discuss how they are related by presenting an extensive review of mathematical and computational proposals related to them, and then to explore the implications of this for understanding behavioral and neuroscience data. We argue that opportunities for improved understanding of behavior and its neural basis are likely being missed by failing to distinguish between novelty and surprise.

## 1. Introduction

Novelty and surprise play significant roles in animal behavior and in attempts to understand the neural mechanisms underlying it. They are intimately connected to sensory processing, attention, learning, and decision making. Theories of motivation, particularly of intrinsic motivation (Deci and Ryan, 1985; Baldassarre and Mirolli, 2013), place novelty and surprise among the primary factors that arouse interest and motivate exploratory or avoidance behavior. Novelty and surprise also play important roles in technology, where detecting observations that are novel or surprising is central to many applications, such as medical diagnosis, text processing, surveillance, and security. In many—perhaps most—of these studies, novelty and surprise are not distinguished from one another: the words are used more-or-less interchangeably.

However, while undeniably closely related, novelty is in fact very different from surprise. The ordinary dictionary definition of novelty refers to the quality of not being previously experienced or encountered, while surprise refers to the result of encountering something suddenly or unexpectedly. In the most abstract setting (and ignoring many subtleties with which we attempt to deal below), detecting novelty requires examining (by one means or another) the contents of memory to determine if the stimulus has or has not previously been experienced and attended to. Surprise, on the other hand, is the result of a discrepancy between an expectation and an observed actuality. This comparison of an experience with an expectation does not require examination of the contents of memory despite the fact that an expectation is clearly built on previous experience. Something can be unanticipated without being un-experienced.

To pick just two illustrations of how natural it is to blur the distinction between novelty and surprise, consider the following quotations. Marsland (2003) writes: “Novelty detection, recognizing that an input differs in some respect from previous inputs, can be a useful ability for learning systems, both natural and artificial. For animals, the unexpected perception could be a potential predator or a possible victim.” When discussing what happens when a naked man enters a classroom, Ranganath and Rainer (2003) write: “Suffice to say, the entrance of the naked guy was a novel event in that it was unexpected and out of context.” Although this blurring is completely understandable given how closely related novelty and surprise can be and the difficulty of formalizing the concepts, we argue that the failure to clearly distinguish between novelty and surprise precludes opportunities for improved understanding of behavior and its neural basis.

The purpose of this article is foremost to remind readers of differences between novelty and surprise, to discuss how these concepts are related, and to explore the implications of this for understanding behavioral and neuroscience data. A review of all that has been written about novelty and surprise is significantly beyond the scope of this paper. Here we present an extensive review of mathematical and computational proposals related to surprise and novelty, and we discuss these proposals in terms of our common sense notions. We also point out key factors that distinguish surprise from novelty, and we argue that some of the definitions in common use are misleading, as are some of the labels applied to results of experiments by psychologists and neuroscientists.

A caveat with respect to the interpretation of empirical data is needed. The distinction between novelty and surprise critically depends on the mechanisms in play when the nervous system produces the experimental results in question. As a consequence, it is to be expected that one cannot say with certainty whether experimental results provide evidence for novelty or for surprise when the actual mechanisms implemented by the brain are incompletely known. However, we suggest that by distinguishing novelty from surprise some existing results might be reinterpreted in a way that improves our understanding of behavior and the neural machinery that underlies it. And, even more importantly, keeping the distinction in mind may be a useful heuristic for studying the brain. Although the names used to describe results are not important, the distinction may encourage neuroscientists to ask questions such as: Is there a predictor at play? If so, where is it? What kind of predictions does it produce? On the basis of what information? Or, if there is no prediction, what are the memories that are searched for? Where are those representations stored? These are important questions that may not arise as clearly if one fails to distinguish between novelty and surprise.

This article begins with accounts of representative examples of how the words have been interpreted, first addressing surprise (Section 2) and then novelty (Section 3). For the most part, the examples in each of these sections were chosen because they provide formalizations related to each concept, although not all of them are intended to model surprise or novelty in animals. The examples are placed in either the surprise or novelty section on the basis of which word their adherents chose to associate with them. Section 4 summarizes the main features of surprise and novelty, viewing each in an idealized form that largely ignores the more complicated issues about how they are related. Section 5 takes on some of these issues by examining the relationship between less idealized views of surprise and novelty. Some of the categories in which formalisms were placed in Sections 2 and 3 are reconsidered here. Section 6 considers how an improved understanding of differences between surprise and novelty may have beneficial consequences in neuroscience, where it can serve to sharpen the interpretation of experimental results and raise useful questions for continuing research. The article ends with a brief summary and concluding remarks.

## 2. Surprise

Of the two concepts novelty and surprise, surprise is probably the easiest to characterize. There is wide agreement that surprise is an emotion arising from from a mismatch between an expectation and what is actually observed or experienced (e.g., Ekman and Davidson, 1994). Since our concern here is not with the emotion of surprise but rather with the conditions that elicit it, by surprise we mean these eliciting conditions. Surprise requires a mechanism for comparing an expectation with actuality.

But what is an expectation and how is one aroused? An expectation is usually thought of as a mental representation of a stimulus or event that is aroused by some cue or set of cues that has regularly preceded that stimulus or event in the past. Alternatively, an expectation might be aroused by an inferential process that predicts the occurrence of a stimulus or event (Berlyne, 1960). According to the most straightforward view, expectations are representations of the values that some perceptual features are likely to assume in the future. However, expectations are naturally expressed in probabilistic terms as well, where a probability distribution over the range of possible observations can be considered to be a “belief state,” a kind of expectation that can generate surprise. If an estimated probability of an observation is available to the perceiving agent when the observation is made, then the certainty of the observation can be compared to its probability of occurrence, yielding a measure of surprise. Importantly, expectations as probabilistic beliefs are usually conditioned, in the sense of being conditional on a particular state or context. This notion of expectation (which is not the same as the expectation, or expected value, in probability theory) underlies Bayesian views of surprise that we discuss in Section 2.2 below.

The psychologist D. E. Berlyne, who wrote extensively about novelty, surprise, and curiosity, used the term *incongruity* for the situation of a stimulus creating an expectation that is unfulfilled by other stimuli that occur at the same time (Berlyne, 1960). The “two-headed lady” of his example is incongruous because her extra head violates the expectations generated by the rest of her image. Berlyne regards this as a special case of surprise that does not involve the passage of time, while acknowledging that it might actually involve time because parts of the incongruous stimulus may be scanned in succession.

Surprise plays a key role in theories of learning and finds natural expression in the framework of Bayesian statistics. Here we first discuss how prominent models of associative learning represent expectations and surprise, followed by a description of a modern Bayesian theory of surprise in which expectations appear as probability distributions over classes of environment models. Then we briefly discuss closely related information-theoretic notions of surprise. We discuss these examples in some detail because they are concrete examples of how surprise has been expressed in formal terms.

### 2.1. Surprise in associative learning theory

Surprise plays a key role in theories of classical, or Pavlovian, conditioning. In classical conditioning experiments, conditioned stimuli (CSs) are followed after a short time by biologically significant events (such as a shock, food, etc.), called unconditioned stimuli (USs) that reflexively produce unconditioned responses (URs). Great care is taken to prevent the animal's response to the CS from influencing the occurrence of the US (unlike instrumental conditioning experiments where a reward is contingent on the animal's behavior). After repeated trials consisting of the CS-US sequence, the animal comes to produce a conditioned response (CR) that resembles the UR but occurs as a response to a CS. For example, an air puff to the eye (the US) elicits a reflexive eye blink (the UR). When regularly preceded by another stimulus (the CS), say a tone or a light, occurrence of the CS comes to elicit an eye blink that anticipates the US. The process is often regarded as one of learning about predictive relationships among stimuli.

What is now called Kamin blocking is the failure of an animal to learn to elicit a CR when a CS is presented to an animal as part of a compound that includes another CS that had been previously conditioned to elicit a CR (Moore and Schmajuk, 2008). Kamin thought that this might be due to the fact that the US is no longer surprising since it is already predicted by the previously conditioned CS:

… perhaps, for an increment in an associative connection to occur, it is necessary that the US instigates some mental work on the part of the animal. This mental work will occur only if the US is unpredicted, if it in some sense surprises the animal. Thus, in the early trials of a normal conditioning experiment, the US is an unpredicted, surprising event of motivational significance and the CS-US association is formed. (Kamin, 1969, p. 293)

This idea that an organism learns only when events violate its expectations, that is, when the organism is surprised, was elaborated by Rescorla and Wagner in the most widely-known and influential model of classical conditioning (Rescorla and Wagner, 1972):

The central notion here can also be phased in somewhat more cognitive terms. One version might read: organisms only learn when events violate their expectations. Certain expectations are built up about the events following a stimulus complex; expectations initiated by that complex and its component stimuli are then only modified when consequent events disagree with the composite expectation. (Rescorla and Wagner, 1972, p. 75)

In the associationist tradition, the Rescorla-Wagner model adjusts associative strengths of stimuli that specify how strongly each stimulus predicts the US. Each constellation of stimuli that occurs (CS) generates a *composite expectation* for the US. This composite expectation is the weighted sum of the saliencies of the stimuli in the constellation, each weighted by its corresponding associative strength for the US. The model adjusts the associative strengths that specify how strongly each component *cs_i_* of the CS present on a trial predicts the US:

(1) $$\Delta V_{cs_{i}}=\alpha_{cs_{i}}\beta (\lambda -V),$$

where *V*_*cs*_*i*__ is the associative strength of component *i* of the CS and Δ*V*_*cs*_*i*__ is its change, α_*cs*_*i*__ is the salience of component *i* of the CS, β is the learning rate parameter associated with the US, λ is the asymptote for learning for the US, and *V* is the composite expectation for the CS. The model adjusts the associative strengths of the stimuli present on each trial up or down depending on λ − *V*, the difference between the composite expectation, *V*, and the associative strength supported by that particular US, λ, which we call the “target associative strength.”

For the sake of brevity we skip further details and the important role this model has played in the history of animal learning theory (see Schmajuk, 2008, for a review; see also Lepora et al., 2010, and Mannella et al., 2010, for two models that capture the basic brain mechanisms with which classical conditioning is implemented in, respectively, cerebellum and amygdala). The key point is that the difference, or discrepancy, λ − *V*, is considered to be a measure of *surprise*: a constellation of stimuli generates an expectation that is compared with what actually happens.

The Rescorla-Wagner model is an example of an error-correcting learning rule such as the Widrow-Hoff Least Mean Square learning rule (Widrow and Hoff, 1960) and the well-known error backpropagation algorithm (Rumelhart et al., 1986) where the US corresponds to the “teaching input” or “desired output,” and λ − *V* is the error guiding learning (although the error is sometimes called a teaching signal in biological models of classical conditioning, e.g., Lepora et al., 2010). Error correction is also central to the widely-used Kalman filter and related algorithms, where the error is called the “innovation” or “measurement residual” (Welch and Bishop, 1995).

Connecting the Rescorla-Wagner model to probabilistic notions of surprise is the observation that in the case where the US is represented by a binary variable with values 0 or 1, the model computes the conditional probability of the US given possible patterns of CSs (Dayan and Long, 1998). In addition, the process of error correction is related to Bayesian learning as we discuss in Section 2.2 below.

Error correction is also the basis of Temporal Difference (TD) learning (Sutton, 1988), where the error incorporates information about the *long-term* expectation of reward and not just the immediate reward. TD learning is the basis of a model of classical conditioning that elaborates the Rescorla-Wagner model (Sutton and Barto, 1990) as well as the reward-prediction-error hypothesis about the phasic activity of dopamine producing neurons in the brain (Barto, 1995; Houk et al., 1995; Schultz et al., 1997; Schultz, 1998). TD learning is not restricted to predicting reward; the role of reward can be replaced by other stimulus features, and it can be generalized to networks of interrelated predictions (Sutton and Tanner, 2004).

In accord with the associationist view, the associative strengths of the stimuli needed for determining a composite expectation become available as a consequence of the mere occurrence of the stimuli. They have been formed in response to the animal's experience over time in observing sequences of stimulus constellations. Think of a two-layer neural network whose connection weights from its input layer to its output layer correspond to the adjustable associative strengths. In response to input patterns the network computes composite expectations in the form of the activity levels of the output units. Target output values representing USs, provided by so-called “teaching inputs,” are compared to the network's actual outputs—the surprise computation—to determine the error that drives learning. In addition to participating in this comparison, these expectations also directly determine the strength of the animal's tendency to produce a CR.

This process does not require a scanning of the organism's memory for previously experienced instances of the stimulus constellation that is currently present: this experience has been cached in the connection weights, and the network reads out an expectation in response to the current input pattern. In a neural network setting that considers the relative timing of inputs (i.e., the teaching input is whatever stimulus pattern occurs shortly *after* the input pattern setting the activation levels of the input units), the network becomes a *predictor*, meaning that each of its output patterns will tend to resemble the input pattern that comes next. (Of course this assumes the network is complex enough to represent the prediction function.) The process is not tied to a specific US. The network's weights summarize, in a statistical sense, the totality of the organism's previous experience as to what stimulus constellations tend to follow other stimulus constellations. In machine learning, one would say that a *forward model* of environmental contingencies is learned via *supervised learning* (Barto, 1990).

Other concepts have been proposed for how an expectation for associative learning might be implemented in the nervous system. For example, Grossberg (1982) proposed that an expectation is a feedback pattern of neural activity derived from signaling across an entire network gated by long-term memory, and that unexpected events trigger a “mismatch-modulated arousal burst,” i.e., what we would call a surprise signal.

### 2.2. Bayesian surprise

A formal theory of surprise was proposed by Itti and Baldi based on the Bayesian framework (Itti and Baldi, 2005, 2006, 2009). In this framework, probabilities, which correspond to subjective beliefs, are updated as new observations are made using Bayes' theorem to convert prior beliefs into posterior beliefs. What they call *Bayesian surprise* is a measure of the difference between an observer's prior and posterior beliefs.

Here is how they formalize this. An observer is assumed to have background beliefs characterized by a prior probability distribution over hypotheses or models of its world, *M*, that are in some space of models, 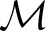: 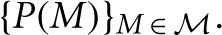


Upon obtaining new data *D*, the observer updates this prior distribution into the posterior distribution by applying Bayes' theorem: 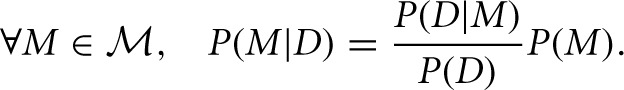


Bayesian Surprise is a measure of the dissimilarity between the prior and posterior distributions. Itti and Baldi do this using the relative entropy, or Kullback-Leibler (KL) divergence, between these distributions: 
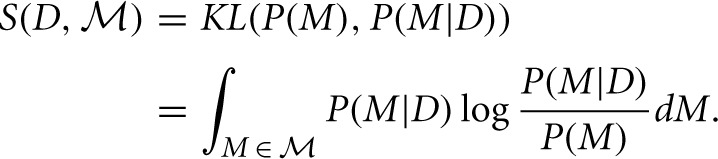


This measure gives the amount of information needed to transform the prior into the posterior distribution:

A unit of surprise—a “wow”—may then be defined for a single model *M* as the amount of surprise corresponding to a two-fold variation between *P*(*M*|*D*) and *P*(*M*), i.e., as log *P*(*M*|*D*)/*P*(*M*) (with log taken in base 2), with the total number of wows experienced for all models obtained through the integration [in the equation above]. (Itti and Baldi, 2009)

According to this theory, surprise is a measure of the discrepancy between beliefs before and after an observation. A surprising event is one that is not well predicted by the animal's current beliefs formed in response to its previous experience. In this case, the expectation that determines surprise is the set of beliefs held by the agent before the observation in question, that is, the prior probability distribution over possible world models: {*P*(*M*)}_*M* ∈ 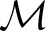
_. Itti and Baldi (2005, 2006, 2009) argue that this definition has key advantages over alternatives in being more principled, more widely applicable, and more able to account for what attracts human visual attention. Importantly for our purposes, these authors also discuss how assessing surprise differs from detecting statistical outliers, which is one of the notions commonly (though erroneously we will argue) invoked for detecting novelty. We discuss this in Section 5 where we examine differences between surprise and novelty.

Schmidhuber and colleagues (Schmidhuber et al., 1994; Storck et al., 1995) proposed using Bayesian surprise (as later defined by Itti and Baldi) as a measure of learning progress for reinforcement learning agents. This measure of surprise generates a “curiosity reward” that encourages the agent to behave so as to continue learning efficiently by seeking regions of its environment where it is surprised while avoiding regions where it is “bored,” either because it has already learned as much as it can in those regions (thereby eliminating surprise) or because there are no learnable regularities (so that surprise is absent because new information is not acquired). This is one of the first proposals for how ideas related to what psychologists call intrinsic motivation can be implemented in a machine learning system, and much additional research has been lately done in this area (Baldassarre and Mirolli, 2013).

Itti and Baldi (2005, 2006, 2009) were concerned with attention rather than learning, but their concept of surprise arises from the Bayesian approach to learning where a prior belief distribution is updated by Bayes' theorem to a posterior distribution upon each new observation. Large Bayesian surprise means that learning from a new observation has made a large change in the animal's beliefs about the contingencies in its world. In its most general form, Bayesian learning does not explicitly involve the computation of prediction errors. Instead of processing errors generated by an existing model, learning processes evidence for all possible models and updates beliefs accordingly. Unlike error-correction learning, where the error as a measure of surprise is the direct driving force of learning, Bayesian surprise is the result of learning but not its direct cause, coming after the Bayesian update instead of before it.

However, Bayesian learning can be approximated, and in some cases computed exactly, by an error-correction process. The Kalman filter, for example, uses error correction to perform Bayesian learning in the context of linear-Gaussian systems (Welch and Bishop, 1995). The mean of the Gaussian posterior distribution is updated by multiplying the innovation, or prediction error, by the Kalman gain which controls the allocation of weight between the prediction of a current model and a new observation based on a measures of confidence in the model and in the observation. Bayesian learning can be approximated in a number of ways, such as through the Laplace approximation and variational methods (Bishop, 2006), that permit updates to be made on the basis of prediction errors. Variational approximation plays a key role in the hierarchical architecture proposed by Mathys et al. (2011), who discuss the relationship of the resulting learning process to error-correction methods like the Rescorla-Wagner model.

Models of classical conditioning based on Bayesian methods, including the Kalman filter, have been proposed that go beyond the account provided by the Rescorla-Wagner model (Dayan et al., 2000; Kakade and Dayan, 2002a; Courville et al., 2004, 2006). Changes in the world, and therefore changes in the correct world model, are sources of Bayesian surprise. Bayesian methods not only update beliefs in specific models but also the confidence in those beliefs, and surprise causes *decreased confidence* in current beliefs. As a result, new observations should be given more weight than previous observations (as in the Kalman filter), implying that the speed of learning about the uncertain predictive relationships should increase. This Bayesian account of increases in the rate of animal learning observed in certain experiments (Rescorla, 1971) accomplishes what the Pearce-Hall model model (Pearce and Hall, 1980) does via its use of an explicit measure of surprise as the magnitude of a prediction error. TD learning has also been developed in a Bayesian framework (Engle et al., 2003).

Another area in which prediction errors appear in a Bayesian framework is in the “predictive coding” architectures of Rao and Ballard (1999) and Friston and Kiebel (2009). These are layered hierarchical systems going from input levels to levels encoding information in a more abstract fashion. The key aspect of these systems is that the bottom-up flow of information from sensations to abstract representations is paralleled by a top-down information flow where the top levels project predictions to the lower-levels. This allows higher-level stages to receive information only through the information mismatch between their predictions and sensations, so that higher levels receive only unpredicted information. Prediction errors are used to propagate information from the bottom up to the higher levels of the system, and also to continuously update the top-down predictors. These proposals refine the concept of surprise as they capture surprise at multiple levels, namely from the prediction of simple, isolated events at the lower levels, to the prediction of the behavior of more complex compounds of items at the higher levels.

### 2.3. Information theoretic surprise

Although Itti and Baldi's Bayesian theory of surprise is connected to information theory (KL divergence is a measure of information gain), other concepts of surprise are more directly based on information theory. One example is what Tribus (1961) called *surprisal* to refer to the self-information of the outcome of a random variable, which is a measure of the information content of the outcome. If outcome ω occurs with probability *P*(ω), then the self-information, or surprisal, is − log *P*(ω). Thus, an outcome that is highly unlikely has high surprisal when it occurs. The expected value of surprisal for observations drawn from a random source is the entropy of that source. Computational linguists, e.g., Roark (2011) and Monsalve et al. (2012), use the term *lexical surprisal* to refer to the negative log of the *conditional probability* of a word in a sentence given the preceding words in the sentence. Although Tribus' definition of surprisal does not explicitly invoke conditional probabilities, there is always an implicit assumption that surprisal is conditioned on a context or model. Therefore, when we refer to surprisal below, we always have a conditional form of surprisal in mind.

An important contrast can be drawn between surprisal and Baysian surprise. The usual example is to consider viewing a television screen showing white noise, or “snow” (Schmidhuber et al., 1994; Storck et al., 1995; Itti and Baldi, 2005). After a while this becomes very boring even though the information content of each frame, or its surprisal, is very high because there are so many equally-likely patterns of random noise. On the other hand, a viewer's Bayesian surprise will decrease and eventually disappear as their beliefs adjust so that random frames become expected. “Thus, more informative data may not always be more important, interesting, worthy of attention, or surprising” (Itti and Baldi, 2005).

Tribus' notion of surprisal plays a prominent role in the global brain theory of K. Friston and colleagues which is based on the principle of “free-energy minimization” (Friston et al., 2006; Friston, 2009, 2010). This principle states that intelligent agents aim to minimize a free energy function of their internal states. If one assumes that an agent maintains a model of the causes of its sensory input, this principle implies that intelligent agents act on their environments to avoid surprises, which means working to make observations that conform to their expectations. Another component of this theory is that intelligent agents learn by revising their models to make more accurate predictions. These implications can be seen to follow from free-energy minimization through the perspective of variational Bayesian inference. Free energy (in this case the variational free energy) is always greater than or equal to the negative log of the evidence, or the marginal likelihood, of the agent's model. Model evidence is the probability of observations given the agent's current model: if *s* denotes an agent's sensory state at some time and *M* denotes its current model, the model evidence is *P*(*s*|*M*) (where hidden states have been marginalized out). Thus, acting to minimize this free energy function tends also to minimize the negative log of model evidence (since the latter quantity is always less than or equal to the free energy). This is equivalent to tending to maximize the (positive) log of model evidence, which is the same as tending to maximize the model evidence itself since the logarithm is a monotonically increasing function. The theory's connection to surprisal is due to the fact that the negative log evidence for a model is the surprisal conditioned on that model, −log *P*(*s|M*), so that maximizing model evidence is the same as minimizing this notion of surprise. According to this theory, then, intelligent agents act on their environments to suppress discrepancies between their predictions and what they actually experience, that is to avoid being surprised.

The theory also relates to Itti and Baldi's (and Schmidhuber's) notion of Bayesian surprise. In addition to acting to increase evidence of a current model, agents can reduce free energy by adjusting their model to make more accurate predictions. Through a learning process, a current probability distribution over models (a prior distribution) is updated to a new distribution (a posterior distribution) that takes into account each new observation. As the model becomes more accurate, the KL divergence between these distributions—that is, the Bayesian surprise—decreases, which decreases free energy. The Bayesian surprise becomes zero only when the model makes perfect predictions. An additional implication of this theory arises from the role of model evidence in Bayesian model comparison, where there is an automatic penalty for model complexity. This implies that the work done by agents to increase how well their model accounts for observations is balanced by a tendency to minimize model complexity, a form of Occam's razor. Friston and colleagues present hypotheses about how the brain might implement the elements of this theory (Friston et al., 2006; Friston, 2009, 2010).

In his book “Novelty, Information, and Surprise” Palm (2012) provides definitions of all three of these terms. Roughly, novelty is the same as Tribus' surprisal, but surprise is given an interesting definition that depends on the concept of a “description,” which is a mapping from possible outcomes of a random variable to propositions that are true for a collection of outcomes. A key aspect of this theory seems to be that by knowing the description an observer is using, that is, by knowing the whole mapping, it is possible to consider the probability that an outcome will have the same description as the outcome observed. Then the amount of surprise experienced by an observer depends not on the probability of the observation, but on the probability of any observation with the same description. Palm gives the following example. Suppose that in a state lottery the sequence of numbers (1, 2, 3, 4, 5, 6) were to be drawn. This would be much more surprising than the sequence (5, 11, 19, 26, 34, 41) even though both sequences have the same probability of being drawn. “The reason for our surprise in the first case seems to be that this sequence can be exactly described in a very simple way: it consists of the first six numbers. … it is much more probable to obtain a sequence that does not admit a simple exact description …. In the special case of (1, 2, 3, 4, 5, 6) we could argue that there are only two such extremely simple sequences, namely the last 6 and the first 6 numbers” (Palm, 2012, p. xix). Palm argues that his extension of classical information theory allows one to incorporate a “person's interests, intentions, and purposes.” How this intriguing view of surprise relates to the more familiar ones discussed above is not yet completely clear to the authors.

### 2.4. Summary

According to the commonsense notion as well as the most prominent formulations, surprise involves a comparison between an expected and an actual observation. The comparison does not need to entail a scan of the contents of memory. Expectations formed on the basis of past experience can be linked directly to stimuli so that they are aroused by the occurrence of those stimuli, or aroused by an inference process in the absence of those stimuli. Surprise is a measure of the discrepancy this comparison reveals, whether it is a simple signed difference as in error-correction learning rules, the KL divergence in Itti and Baldi's Bayesian surprise, or some other measure. Predictive coding by hierarchical systems suggests how surprise might be generated at different levels of abstraction. The term surprisal has been proposed for an observation's self information, a quantity inversely related to the probability of the observation conditional on a model. Bayesian surprise and surprisal differ in significant ways. Friston's global brain theory based on the free-energy principle suggests that intelligent agents act in order to reduce surprisal conditioned on their current models, while they also reduce (future) Bayesian surprise by adjusting their models to make better predictions.

## 3. Novelty

Confronting the problematic concept of novelty, Berlyne (1960) emphasized a number of relevant distinctions. First, he distinguished between *short-term*, *long-term*, and *complete* novelty. Something may never have been encountered before (complete novelty), or not encountered in the last few minutes (short-term novelty), or not encountered for some intermediate time, e.g., a few days (long-term novelty). Another distinction is that between *absolute* and *relative* novelty. A stimulus is *absolutely novel* when some of its features have never been experienced before, whereas it is *relatively novel* if it has familiar features but they occur in some combination or arrangement that has not been previously encountered.

Berlyne claimed the following:

Any new experience, even if it does not seem to be a combination of familiar experiences, must have some definite degree of resemblance to experiences that have occurred before. It will inevitably be possible to insert it into an ordering of familiar stimuli or to assign to it values among dimensions that are used to classify them. (Berlyne, 1960, p. 19)

He gives the example of seeing a man taller than any seen before: it is still possible to place the experience on a familiar scale, or more generally, to locate the experience in the appropriate multidimensional feature space. Further, according to Berlyne:

For any adult human being, or even any adult dog, cat, or rat, a new stimulus must be similar to, and relatable to, a host of familiar and frequently experienced entities. However, bizarre a non-sense figure may be that is shown to a human adult, it must consist of lines, angles, and curves such as he has seen on countless occasions. (Berlyne, 1960, p. 20)

Note that Berlyne restricts this comment to adults. The situation must be different for young children, due not only to their relative lack of experience but also due to the deeper need to establish the feature spaces and dimensions that are useful for categorizing experience. For designers of artificial agents this is a key issue.

Berlyne's distinctions are important because they connect to our ordinary understanding of what the term novelty means while revealing some of the issues that make the concept problematic. In formal notions of novelty to which we now turn, the links to our commonsense notion are not always apparent.

### 3.1. Memory-based novelty

The simplest translation of our commonsense idea of novelty into a more precise notion is that the novelty of an event is assessed by examining a memory store of past observations, where a memory system might require more than one experience of an event to form a lasting memory. An observation is completely novel, to use Berlyne's term, if a representation of it is not found in memory. If memory fades with time, this process assesses short-term or long-term novelty depending to the fading rate. This of course ignores many aspects both of novelty and of memory, and it may not be feasible from a computational perspective.

But some more sophisticated methods for novelty detection are elaborations of this basic idea. Novelty detection based on clustering is one example. Using a distance measure based on similarity, data can be clustered into classes so that items in a class are “close” to one another and not close to items in the other clusters. Novelty here means that an item is not close enough to the mean of an already existing cluster, so that a new cluster needs to be formed. There are very many clustering methods, and there are many methods for determining when a new cluster should be added (Markou and Singh, 2003).

Determining distances from existing clusters is a search of a memory that stores the cluster means, making it more feasible than a naive memory-based method. Prominent neural network methods for novelty detection, such as methods based on self-organizing feature maps (Kohonen, 1984; Nehmzow et al., 2013), perform this basic process where the memory scan is performed in parallel by the network. Of current interest in statistics and machine learning are Bayesian non-parametric clustering methods (Gershman and Blei, 2012). Instead of specifying the number of clusters in advance, these methods allow the number of clusters to grow as new data items arrive. These methods do not involve a literal scan of memory, but determining whether a new cluster is needed essentially relies on determining that none of the existing clusters properly explains the data.

Another kind of memory-based novelty arises in the case of content-addressable associative memory systems. Perhaps the most well-known and simplest is the correlation matrix memory proposed by Kohonen (1977, 1980, 1984). Instead of being stored in separate memory locations, information is superimposed and distributed across a memory substrate, for example a neural network, and retrieval is a kind of filtering process. The stored items are vectors of real numbers, and the memory is a matrix formed from the stored vectors in such a way that upon being presented with an input vector, the system produces as output a weighted sum of all the stored vectors, where each weight is a measure of how well that stored vector correlates with the input vector. When the input vector is a distorted version or a fragment of a stored vector, it is expected that it will correlate most strongly with that vector and much less with the other stored vectors, implying that the memory's output will be a less noisy version of the input vector or a “completion” of it. Mathematically, the memory's output is the orthogonal projection of the input, *x*, onto the subspace, 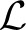, spanned by the stored vectors, which is the vector, call it $\hat{x}$ ∈ 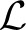 that is “closest” to *x*. Every vector *x* can be expressed as the sum of $\hat{x}$ and a vector, $\tilde{x}$, in the subspace orthogonal to 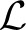. Kohonen (1977) says that “$\tilde{x}$ is the amount that is ‘maximally new’ in *x*. It may be justified to call this component the ‘novelty,’ and the name Novelty Filter is hereupon used for a system which extracts $\tilde{x}$ from input data *x*” …. Roughly, then, this kind of novelty refers to those fragments or aspects of an observation that are not fragments or aspects of previously stored experiences. Our memory systems are undoubtedly much more complicated than a correlation matrix memory, but it is worth keeping this example in mind when we discuss associative novelty as studied in neuroscience in Section 6.2 below.

### 3.2. Novelty as statistical outlier

A common notion is that an observation is novel if it is a *statistical outlier*, meaning that it is significantly different from other members of the sample from which it is drawn. In general terms, detecting outliers requires modeling the usual distribution of observations and detecting when an observation departs significantly from the model. Sometimes this is called *anomaly detection*. Many methods have been proposed to detect outliers and to handle them, but what concerns us here is what being an outlier means with respect to our common idea of novelty and how it differs from surprise.

One area in which this idea of novelty plays a prominent role is machine learning. For example, learning a classification rule by supervised learning involves adjusting a classifier's parameters on the basis of training examples drawn from a corpus of labeled examples. It is important that the corpus of training examples is representative of the input data to which the classifier will be applied. Novelty detection for supervised learning is the problem of determining if an input does not belong to the class of inputs represented by the training examples, i.e., determining if the input is an outlier. For novel inputs, the output of the classifier will be considered unreliable.

Nearly all the statistical approaches to this problem model the probability density of the training data and identify inputs as novel if they fall in regions of low estimated density. Many methods exist for estimating probability densities from a finite number of samples, both parametric or non-parametric (Duda and Hart, 1973; Markou and Singh, 2003), and many methods have been suggested for how to use the estimated probabilities to determine when an input should be regarded as novel. The details of these methods need not concern us here; the principle remains the same: according to this view *novelty means having a low estimated probability of occurrence*. Note that according to the definition of surprisal given in Section 2, this is the same as saying that being novel means having high surprisal, a point to which we return in Section 5 below.

We commented in Section 2.3 that although Tribus' definition of surprisal does not explicitly invoke conditional probabilities, there is always an implicit assumption that surprisal is conditioned on a model or context. Estimated probabilities for outlier detection are conditioned on the context of the collection of samples and background assumptions about the sample space. This raises questions about equating novelty with “low probability” because it is based on the assumption that the system can represent the entire domain of possible samples in advance of experiencing them, and so can assign zero probability to all instances not observed up to a given moment. An aspect of our commonsense notion of novelty for which this view is not able to account is the possibility that an observation might occur that the system is not able to represent in terms of existing categories. Assuming that the sample space consists of all possible configurations of the lowest-level sensor readings may be a solution for artificial systems (e.g., the pixels of a camera), but it seems an inadequate account of biological memory which is typically not so eidetic. Indeed, as we discuss in Section 6 below, novelty may trigger brain activity whose function is to acquire new representations.

### 3.3. Summary

Berlyne (1960) distinguished between several difference senses in which the term novelty is used, and formalizations of novelty are not as unified as those of surprise. Straightforward interpretations involving searches of memory for previous encounters do not do justice to the complexity of either the concept of novelty or of the nature of memory. Clustering-based concepts expand naive memory search and make better contact with the commonsense notion of novelty as the quality of being different from what is in a memory store. Content-addressable associative memory systems suggest a more abstract notion of novelty as, roughly, fragments or aspects of an observation that were not present in previous experiences. Statistical interpretations in terms of outlier detection have many applications, but as we argue below they also abstract away from important aspects of our commonsense understanding. In neuroscience additional categories of novelty are described, which we discuss in Section 6.

## 4. Novelty and surprise: typical features

We have seen that there are various proposals about how to define surprise and novelty, all having some strengths. On this basis, we think it is premature to propose definitive definitions. Nevertheless, we also think it is possible and useful to highlight the main features of the two concepts that represent the “poles” around which the different definitions should gravitate. Table 1 displays these features, and we now briefly explain them.

**Table 1:** **The typical features of novelty and surprise**.

**Features**	**Novelty**	**Surprise**
Type of knowledge store, process involved	Memory, memory recall	Predictor, prediction
Variants of the knowledge and process involved	- Formation of new representations	- Deterministic expectations
	- Formation of new links between the representations of the features/components of the novel data	- Stochastic expectations
Time	Time not a key factor: items in memory are always available for comparison	Incoming data usually compared with a temporalized prediction
Processes for novelty/surprise triggering	One phase:	Two phases:
		- Formulation of prediction
	- Experience does not match memory	- Prediction is violated
Typical functions	- Support the formation of new representations	- Support the improvement of predictions
	- Generate learning signals for the sub-component detecting novelty, or for other sub-components	- Generate learning signals for the predicting sub-component or for other sub-components
	- Direct/motivate attention and learning resources to novel stimuli	- Direct/motivate attention and learning resources to unpredicted stimuli

A key difference between novelty and surprise is due to the type of knowledge store they use and the way they process such knowledge. Novelty is based on memory stores and the processes that determine if a given item is, or is not, in the store. Surprise, on the other hand, is based on expectations of systems capable of predicting, the processes generating such expectations, and the processes that compare the expectations with what is actually experienced. An observation is novel when a representation of it is not found in memory, or, more realistically, when it is not “close enough” to any representation found in memory. Novelty triggers the formation of new representations for entry into long-term memory. These representations can then be exploited to perform other cognitive processes, including the generation of surprise by exploiting already existing representations (Lisman and Grace, 2005; Kumaran and Maguire, 2007). The case of surprise is different because its core element is not the incoming item but the predicted item. Indeed, the incoming item can be either familiar or novel—this does not count. What counts for surprise is that the system perceives “something” that is different from the prediction, whatever that “something” is.

Novelty and surprise also differ with respect to their relation to time. The expectations or predictions that underly surprise have to do with the dynamic flow of events happening in time (with the possible exception of spatial predictions underlying Berlyne's notion of incongruity, which may, however, involve the visual scan of a stimulus, thereby again involving time). Predictions typically involve a specific time, or range of times, in the future when something is expected to happen: “If I see A at time *t*, then I expect to see B at time *t* plus something.” Novelty, on the other hand, seems not to be strictly related to time. The comparison of current experience with the contents of memory, i.e., the process that supports novelty detection, is not sensitive to the time at which a memory was formed, nor to the time the novel item is perceived: what really matters is only the absence of a representation of the perceived stimulus in memory. Berlyne's distinction between short-term, long-term, and complete novelty refers to differences in how this process may work, but in none of these cases is the timing of the perception as critical as it is for surprise.

Both surprise and novelty increase an animal's level of arousal, direct its attention, enhance learning, and elicit other appropriate behavior. But in some other respects surprise and novelty differ in their typical functions. Where novelty often supports the acquisition of representations, surprise supports the improvement of predictions. More specifically, novelty supports the acquisition of items by memory, while surprise plays a key role in improving the capacity of the system to predict (as in error-correction learning reviewed in Section 2.1) or to signal that such an improvement has taken place (as in the Bayesian account as discussed in Section 2.2).

## 5. Relationship between surprise and novelty

Surprise often—perhaps always—accompanies novelty, which may be a major reason the two concepts tend to be confounded. Indeed, if one assumes that an agent is always making predictions about what it is going to soon experience, encountering something novel should not only trigger a novelty response, because no representation has been found in memory that corresponds to the perception, but also surprise, because the agent's expectations must be violated by the novel item which could not have been predicted. In this case, the agent is not predicting that it will not observe that item, but it is predicting that it will observe something else—a prediction that is violated. Whether or not this argument is convincing depends upon whether animals are always expecting something, which in turn depends on what it really means to expect something, which we will discuss shortly.

On the other hand, it is clear that surprise does not imply novelty. A familiar observation may be surprising in a context in which something else is expected. It is easy to come up with examples: for instance, we can be surprised at finding our car door locked when we thought we had just clicked the unlock button on the key fob.

A more interesting example is provided in a study by Huron (2004) of laughter in listeners to Peter Schickele's PDQ Bach compositions. In this example, the expected “something else” is in fact rare, whereas the actual observation is familiar, though unexpected. Schickele has composed a large number of humorous pieces attributed to the fictional P.D.Q. Bach. Huron argues that a plausible explanation for the laughter these compositions induce is that laughter occurs at “dramatic violations of expectation.” In one composition (*Quodlibet for Small Orchestra*), Schickele reproduces a well-known theme from a Beethoven symphony, but instead of continuing with Beethoven's finish to the movement “which is the rarest continuation in Western music with a probability of less than 0.007,” he switches to a “musically banal” conclusion. Invariably, listeners burst into laughter at the moment of this switch. Huron (2004) summarizes:

In short, Schickele's transgression here is a violation of veridical expectation (“That's not how the music goes.”) rather than a schematic transgression (“That's not what happens in music.”) The violation is amplified by the extreme contrast between veridical and schematic probabilities. (Huron, 2004, p. 702)

What Huron means by a “veridical expectation” is an expectation created through past experience with the specific music in question, in this case Beethoven's symphony, which—during listening—generates an expectation for its usual ending. But the usual ending is rare in music in general, that is, its probability of being heard is very low, whereas Schickele's ending has much higher probability. Therefore, the “schematic transgression” is a mismatch between an expectation for something unlikely and the receipt of something familiar.

As discussed above in Section 3.2, a common formalization of novelty in machine learning is that being novel means being a statistical outlier, and novelty detection is accomplished by modeling the probability density function of possible observations and regarding an observation as novel if it falls in a region of low enough estimated density (according to a given threshold or a more sophisticated criterion). We are not aware of claims that this formalization of novelty provides a good account of what novelty means for an animal, but it is pertinent to ask if this notion of novelty is consistent with either our commonsense understanding of the term or novelty's typical features. The answer has to be no. It is true that if the probability of an event occurring is low, then the probability that a representation of that event is stored in memory is low as well. But it is clearly missing something important about novelty to equate low estimated probability of occurrence with novelty. It is easy to think of examples of events that are not novel at all but that have a very low probability of occurring. For example, any event that occurred only once in the past, and that is distinctly different from other experienced events, will likely be assigned a low probability of occurring again. But that event may be vividly memorable and therefore familiar if it were to happen again. Furthermore, if so-called novelty detection happens as a result of a mismatch between one's estimated probabilities and current perceptions, this seems to be a clear case of surprise rather than novelty, as discussed in Section 2. Thus, while treating low probability events as novel may be a good method for machine learning, it is a poor model of what novelty really is and represents a misleading use of the term.

The same reasoning explains why Tribus's term surprisal (Tribus, 1961) is more consistent with what we mean by surprise. Indeed, the surprisal value of an observation, that is, a measure inversely related to its probability of occurring, can be thought of as the discrepancy between its probability of occurring and the fact that it actually occurred. Thus, surprisal appears to be consistent with the notion of surprise according to our analysis (despite the fact that it is basically the same as novelty according to the statistical outlier view of novelty). Surprisal is particularly consistent with our characterization of surprise when it is explicitly conditioned on a context as in the lexical surprisal of computational linguists (Monsalve et al., 2012; Roark, 2011). In this case, surprise as surprisal is triggered by an event occurring in a context in which the estimated probability of its occurrence is low.

Itti and Baldi's (2005, 2006, 2009) Bayesian surprise is not a misleading use of the term since their definition is based on a discrepancy between beliefs before and after an observation. The degree of surprise generated by an observation depends on how strongly it changes the probability distribution over models that characterize an observer's beliefs about how its world works. It is not clear that the Itti/Baldi notion is the only, or the best, Bayesian account of surprise, but this account of surprise is consistent with what we regard as its typical features.

Bayesian surprise has interesting implications with respect to the view of surprise as surprisal. Here is a slightly modified version of an example given by Itti and Baldi. Consider incoming data, *D*, that has a very low probability given the current context *C*, that is, *D* is surprising in the sense of having high surprisal. Suppose the observer has only two models, and the observation has a low probability given the context and either model, that is, *P*(*D*|*C*, *M*_1_) and *P*(*D*|*C*, *M*_2_) are both low. In this case, even though the surprisal of *D* is high, Bayesian surprise would be very low since *D* has little effect on the agent's beliefs: it is not useful in discriminating between *M*_1_ and *M*_2_. This is a very hypothetical example, but it raises the question of which account of surprise is more consistent with the processes that generate surprise in animals.

## 6. Surprise and novelty in neuroscience and cognition

This section considers some important threads of neuroscience research related to surprise and novelty. Enlisting the concepts developed in the previous sections shows how existing results might be reinterpreted in a way that improves our understanding of behavior and the neural machinery underlying it. The goal here is not to cover the large neuroscience literature related to novelty and surprise, but rather to show how keeping the distinction in mind may be a useful heuristic for isolating interesting problems and seeking answers to questions about how surprise and novelty are processed in the brain. Thus, below we focus on a selection of biological cases that involve mechanisms where the distinction between novelty and surprise is blurred or controversial, while omitting consideration of other brain phenomena more reliably associated to each of the two concepts (e.g., cerebellum, forward models, prediction errors, classical conditioning; anterior cingulate cortex, anticipations, error-related negativity; amygdala, classical conditioning).

Modern neuroscience literature distinguishes between three types of novelty to which the brain responds: stimulus novelty, contextual novelty, and associative novelty (Ranganath and Rainer, 2003; Kumaran and Maguire, 2007). These three types of novelty are investigated with different experimental paradigms, involve partially overlapping networks of brain areas, and are based on various neural mechanisms. In addition, an important thread of neuroscience research deals with what have been called dopamine “novelty responses.” In what follows we discuss these four novelty categories in turn, trying to clarify whether the term “novelty” is an appropriate label or if the investigated phenomena have more to do with surprise.

### 6.1. Stimulus novelty

Stimulus novelty refers to the phenomenon for which the neural and behavioral responses to a particular stimulus (e.g., the sight of an object) change when it is experienced multiple times. A typical observation is that with repetition of a stimulus the neurons responding to it present a progressively decreasing activation, a phenomenon called *repetition suppression* (Ringo, 1996; Henson and Rugg, 2003). Repetition suppression is stimulus specific and has been observed in various types of experiments, from classification (Sobotka and Ringo, 1994) to delayed-matching-to-sample tests (Li et al., 1993). Some of the areas most sensitive to the novelty of stimuli are inferotemporal cortex (Ranganath and Rainer, 2003), an area involved in object recognition, the perirhinal cortex (Brown and Aggleton, 2001), an area close to the hippocampus and involved in episodic memory, and the prefrontal cortex (Asaad et al., 1998), the highest multimodal associative cortex.

Stimulus novelty seems to be the classical case of novelty, where the incoming items trigger novelty detection when they do not correspond to any existing memory. The novel items trigger the formation of a neural representation at multiple levels within the brain areas mentioned above, so they progressively became familiar (Ranganath and Rainer, 2003).

An intriguing issue related to stimulus novelty arises from the fact that novel items seem to cause an initial high activation of the brain areas where novelty is presumed to be computed. This raises a twofold question: (a) what are the specific mechanisms that cause such a high activity, and (b) what is its adaptive function? While the question about mechanisms is an interesting challenge for computational modeling, the view that the main function of novelty detection is the formation of representations of the novel items in memory might explain why novel items cause higher activation. Learning often needs to be supported by the production of neuromodulators. The elevated activation caused by novel items might trigger the production of neuromodulators, for example, noradrenaline and achetylcholine (see Ranganath and Rainer, 2003, for a review). In turn, the presence of neuromodulators may support the formation of new neural representations. This hypothesis suggests a number of neuroscientific investigations directed toward understanding the brain mechanisms implementing the various steps of the suggested causal chain, as has already happened with respect to dopamine and hippocampus, which are involved in the other types of novelty detection considered below (see Lisman and Grace, 2005).

### 6.2. Associative novelty

Associative novelty is one of the most subtle and interesting cases of novelty studied in neuroscience. Associative novelty refers to situations where familiar stimuli are associated in novel configurations (Kumaran and Maguire, 2007). The associations can be: *spatial*, where familiar items appear in new spatial locations; *item-item*, where items appear in novel combinations, e.g., two familiar words are paired in an odd fashion; or *temporal*, where familiar items appear in a novel temporal sequence. Interestingly, the field of associative memory is one in which the blurring of the distinction between novelty and surprise is most prevalent. An example is given by the following from O'Keefe and Nadel (1978) with our italics:

Imagine that you are in a classroom … *suddenly*, your attention is diverted when a naked man enters the room. … the entrance of the naked guy was a *novel event* in that it was *unexpected* and *out of context*. … *novel events* attract attention and they are more effectively *encoded in memory* than are *predictable events*.

Associative novelty includes cases that are most difficult to classify, including some that may involve *both* novelty and surprise.

Temporal associative novelty involves a paradigmatic case of surprise: if you perceive a familiar item in a novel temporal sequence, it seems that items that precede the target item constitute the context that supports an expectation which is violated by the appearance of the familiar target item. Hence surprise.

The spatial case is also probably related more to surprise than to novelty. When we perceive a familiar item in a new spatial location, we already have its representation in memory. It is likely that finding the item in a position where we never experienced it just violates our expectation regarding its position—hence surprise. This interpretation is consistent with the fact that in experiments dealing with spatial associative novelty, subjects are typically exposed to the associative pairings for many times before their familiarity/novelty discrimination responses are assessed (Duzel et al., 2003; Kohler et al., 2005). It is most likely that these repeated exposures are needed for expectations to be created, so that they can be violated to trigger the inappropriately-labeled “novelty” signal.

Item-item associative novelty seems to be the more complicated case to classify. To understand whether a case is best called novelty or surprise may require knowing which brain mechanisms are involved. It is well accepted that the hippocampal system is involved in the formation of complex episodic memories and seems to play a critical role for the detection of multiple kinds of novel associations (Wan et al., 1999; Brown and Aggleton, 2001). The *comparator hypothesis* is one of the most established hypotheses about how the hippocampal system detects associative novelty. It refers to the following processes (Hasselmo and Schnell, 1994; Kumaran and Maguire, 2007; Duncan et al., 2012): (a) familiar aspects of the percept (“lures”) actively recall previous memories on the basis of pattern-completion-like mechanisms, for example, an item recalls other items previously experienced in association with it, and (b) some of the perceived items mismatch with the recalled items so that a mismatch signal is triggered. If this theory is correct, than associative novelty is closely related to Berlyne's notion of incongruity, which we classified as a form of surprise in Section 2 because it involves a mismatch between explicit expectations/predictions and incoming data. Kohonen's “novelty filter” (Kohonen, 1977) described in Section 3.1 is relevant to this point: the novelty in an input is, roughly, that part of it that is not predicted by the remaining part. However, it might also be the case that sometimes sets of items are grouped into single compound representations, and that the brain, by searching in memory for these representations and not finding any, registers observation of the set as actual novelty. It is also plausible that in such circumstances both novelty and surprise are simultaneously at play.

The general point here is that some areas of the brain, especially higher-level associative areas such as the hippocampus, may use the same machinery to exploit the representations of associated items to either detect novelty or to detect surprise, depending on the context and the task at hand, and that in some cases both novelty and surprise may be registered. What are the actual mechanisms that the brain uses in each circumstance is an important question for neuroscience research.

### 6.3. Contextual novelty

Contextual novelty is another type of widely-studied novelty, closely related to associative novelty (Ranganath and Rainer, 2003). This refers to the behavioral and neural reactions to stimuli that are familiar but are unexpected given the context in which they occur. Contextual novelty is often studied in *oddball* experiments where, for example, sequences of a repeating auditory stimulus (e.g., a simple tone) are interleaved with rare odd signals (e.g., a “moo” of a cow) (Ranganath and Paller, 1999). The reaction of the brain to an oddball stimulus is often monitored via electric field potentials (Event-Related Potentials—ERP) generated when the brain detects the stimulus. The typical result of these tests is the manifestation of a positive wave of the electric field happening about 200–300 ms after the odd stimulus and named “P300” or “P3” (Friedman et al., 2001). Intense investigation has led to the isolation of a P3a component of the wave, also called “novelty P3” (Soltani and Knight, 2000). Various studies indicate that the novelty P3 originates from a network of brain areas including the the hippocampal system considered above (Soltani and Knight, 2000). This and other elements suggest that overlapping brain machinery might underline associative novelty and contextual novelty (Kumaran and Maguire, 2007).

It is easy to see that in the case of contextual novelty the mechanisms of prediction and surprise, and not of novelty, are in action. Indeed, in the oddball experiments the odd item is appealed to as “novel” even if it is often a familiar item that is presented to the participants in an unpredictable fashion, e.g., a “cow moo” presented after a sequence of simple tones. In this case, the “moo” is surely not novel as the participants have surely heard that sound several times before the experiment. Instead, the “moo” represents a typical example of familiar item that generates surprise because it is unpredicted after a sequence of regular tones. We expect that the clear recognition of what phenomenon is being observed, in this case surprise, will help researchers to recognize new problems and new solutions to them, and to suggest experiments that will lead to a better understanding of the brain processes involved.

### 6.4. Dopamine “novelty” responses

Another important example of the confusion between surprise and novelty can be found in the recent neuroscience literature on dopamine. Dopamine is a neuromodulator that is well known to play a pivotal role in motivational and reinforcement learning processes (Wise, 2004; Berridge, 2007). In the mid 1990s, phasic dopamine activations were recognized to correspond closely with the behavior of the Temporal Difference prediction error (TD error) postulated by the TD algorithm of computational reinforcement learning (Barto, 1995; Houk et al., 1995; Schultz et al., 1997; Schultz, 1998). This has led to the reward-prediction-error hypothesis of the phasic activity of dopamine neurons, which has received a large amount of empirical support and represents one of the most fruitful integrations between computational and empirical research (Ungless, 2004; Wise, 2004; Schultz, 2007; Graybiel, 2008; Glimcher, 2011).

Notwithstanding its success, an important problem faced by the reward-prediction-error hypothesis is that phasic dopamine neuron activity is not triggered only by rewards and reward predictors, but by different kinds of salient stimuli (Horvitz, 2000), such as sudden visual or auditory stimuli that have never been associated with rewards (Steinfels et al., 1983; Ljungberg et al., 1992; Horvitz et al., 1997). Because these responses tend to disappear with repeated stimulation, they have been called “novelty” responses (Schultz, 1998). An interesting explanation of these responses has been proposed by Kakade and Dayan (2002b), who relate them to the problem of exploration: according to these authors, these dopamine activations represent “novelty bonuses” that are generated when an animal perceives novel states and that serve the function of increasing the animal's tendency to explore the environment, thus augmenting the probability that the animal finds rewards. The novelty bonus idea has recently attracted much attention, and it is fostering a number of neuroimaging studies where the activation of the dopaminergic system is studied while subjects are exposed to novel stimuli (e.g., Bunzeck and Duzel, 2006; Wittmann et al., 2008; Krebs et al., 2009).

The problem here is that the so-called novelty responses of dopamine neurons found in animals through electrophysiological studies do not seem to be related to novelty, but rather to surprise. In fact, the stimuli that have been used in those electrophysiological experiments are simple light flashes or sudden sounds, and the dopaminergic responses to lights and tones typically persist after many presentations so that talking about novelty of the stimuli does not seem appropriate (Steinfels et al., 1983; Horvitz et al., 1997; Ungless, 2004). Hence, it is more reasonable to assume that it is the *unexpectedness* of the event, e.g., the sudden appearance of a light or sound, that is responsible for dopamine activation.

Further indirect evidence that the activity of dopaminergic neurons triggered by lights and tones is due to surprise rather than novelty comes from behavioral studies of sensory reinforcement. Sensory reinforcement is the very well-investigated phenomenon that many kinds of sensory events (of which the most frequently studied are again lights and tones) are able to drive the acquisition of instrumental responses. For example, if pressing a bar results in the switching on of a light, an animal will start to press the bar, much as if the bar-press were to lead to a reward such as food (e.g., Kish, 1955; Williams and Lowe, 1972; Glow and Winefield, 1978; Reed et al., 1996). Because we know that dopamine is both necessary and sufficient for appetitive instrumental conditioning (Robinson et al., 2006; Zweifel et al., 2009), it is probably safe to assume that it is phasic dopamine that mediates operant conditioning in sensory reinforcement, just as we assume that it is dopamine that drives standard instrumental conditioning reinforced by food.

Further support that surprise and not novelty supports sensory reinforcement comes from the evidence that light offsets are more-or-less as good reinforcers as light onsets (Glow, 1970; Russell and Glow, 1974). But in the case of light offset, where is the “novel” stimulus that acts as a reinforcer (by supposedly triggering dopamine)? In this case it is even more clear that it is the unexpectedness of the event (surprise), not the novelty of the stimulus (which is absent), that is at play.

We have argued that it is surprise and not novelty that triggers phasic activity of dopamine neurons in animal electrophisiological studies involving lights and tones. But why should this mere misuse of terminology be worth noting? We think there are at least two important reasons to be aware of this misleading labeling. The first reason has to do with the mechanisms underlying phasic activation of dopamine neurons. If one wants to understand how dopamine neuron activity is triggered, it is probably a good idea not to confuse novelty activations due to novel images with surprise activations due to unexpected events. In fact, not surprisingly in human experiments with novel images, it is the hippocampus that seems to be involved (e.g., Lisman and Grace, 2005), whereas light flashes trigger dopamine activity via the superior colliculus, which directly projects to the dopaminergic neurons (Dommett et al., 2005). Furthermore, if it is the unexpectedness of lights or tones that trigger dopamine neuron activity, then the question is raised about the neural circuits providing the predictions that inhibit surprise activations after repeated stimulation. This is a very important question that, to the best or our knowledge, has not yet been addressed. We conjecture that a key reason for this neglect is that these dopamine responses have been regarded as novelty responses, and therefore that they do not involve predictions.

The second reason the novelty/surprise distinction is important with respect to phasic activity of dopamine neurons has to do with the function that these activations play in animal behavior. While it is reasonable to assume that the dopaminergic responses to novel stimuli found in animals are actually “novelty bonuses” that facilitate exploration (Kakade and Dayan, 2002b), it is less reasonable to assume that the same function is ascribed to dopamine activations triggered by unexpected (surprising) events. In fact, it seems more likely that the function of dopamine surprise activations is to encourage the animal to engage in activity to discover which aspects of its own activity may trigger surprising events so that the animal may add new actions to its repertoire (Redgrave et al., 1999; Redgrave and Gurney, 2006; Mirolli et al., 2013).

Finally, to reiterate a point made in Section 2, the TD algorithm, which underlies the reward-prediction-error hypothesis of phasic dopamine neuron activity, is not restricted to predicting reward: the role of reward can be replaced by other stimulus features. The reward-prediction-error hypothesis essentially says that the TD error signals the surprising receipt of reward. But the same machinery equally can signal the surprising receipt of any stimulus. As in the Rescorla-Wagner model, the essence of TD learning is surprise. This adds further support to our suggestion that it would be better to think of the phasic activity of dopamine neurons as responses to surprise rather than to novelty.

## 7. Conclusion

Novelty and surprise play significant roles in animal behavior and in attempts to understand the neural mechanisms underlying it. Surprise and novelty underlie core intrinsic motivations that allow organisms (and promise to allow robots) to acquire useful knowledge and skills in the absence of explicit instruction and externally supplied rewards and penalties. They also play important roles in technology, where detecting observations that are novel or surprising is central to many applications, such as medical diagnosis, text processing, surveillance, and security. The words novelty and surprise are often used interchangeably despite the fact that according to our normal understanding novelty and surprise refer to very different phenomena. Without claiming to do justice to all that has been written about novelty and surprise, we described a sample of past attempts to define these concepts, and we related these definitions to our common sense notions. We pointed out key factors distinguishing surprise from novelty, and we argued that some of the definitions in common use are misleading, as are some of the labels and interpretations applied to results of experiments by psychologists and neuroscientists. But clarifying, indeed in some cases correcting, word usage has not been our goal: opportunities for improved understanding of behavior and its neural basis are likely being missed by failing to distinguish between novelty and surprise.

### Conflict of interest statement

The authors declare that the research was conducted in the absence of any commercial or financial relationships that could be construed as a potential conflict of interest.
